# Exploring Variances of White Matter Integrity and the Glymphatic System in Simple Febrile Seizures and Epilepsy

**DOI:** 10.3389/fneur.2021.595647

**Published:** 2021-04-21

**Authors:** Mustafa Salimeen Abdelkareem Salimeen, Congcong Liu, Xianjun Li, Miaomiao Wang, Martha Singh, Shuqing Si, Mengxuan Li, Yannan Cheng, Xiaoyu Wang, Huifang Zhao, Fan Wu, Yuli Zhang, Habib Tafawa, Anuja Pradhan, Guanyu Yang, Jian Yang

**Affiliations:** ^1^Department of Radiology, the First Affiliated Hospital of Xi'an Jiaotong University, Xi'an, China; ^2^Center for Brain Science, The First Affiliated Hospital of Xi'an Jiaotong University, Xi'an, China; ^3^Department of Radiology, Dongola Teaching Hospital, University of Dongola, Dongola, Sudan; ^4^School of Electronic Engineering, Xidian University, Xi'an, China

**Keywords:** simple febrile seizure, epilepsy, white matter, diffusion tensor imaging, Virchow-Robin spaces, glymphatic system

## Abstract

**Background:** Simple febrile seizures (SFS) and epilepsy are common seizures in childhood. However, the mechanism underlying SFS is uncertain, and the presence of obvious variances in white matter (WM) integrity and glymphatic function between SFS and epilepsy remain unclear. Therefore, this study aimed to investigate the differences in WM integrity and glymphatic function between SFS and epilepsy.

**Material and Methods:** We retrospectively included 26 children with SFS, 33 children with epilepsy, and 28 controls aged 6–60 months who underwent magnetic resonance imaging (MRI). Tract-based spatial statistics (TBSS) were used to compare the diffusion tensor imaging (DTI) metrics of WM among the above-mentioned groups. T2-weighted imaging (T2WI) was used to segment the visible Virchow-Robin space (VRS) through a custom-designed automated method. VRS counts and volume were quantified and compared among the SFS, epilepsy, and control groups. Correlations of the VRS metrics and seizure duration and VRS metrics and the time interval between seizure onset and MRI scan were also investigated.

**Results:** In comparison with controls, children with SFS showed no significant changes in fractional anisotropy (FA), axial diffusivity (AD), or radial diffusivity (RD) in the WM (*P* > 0.05). Decreased FA, unchanged AD, and increased RD were observed in the epilepsy group in comparison with the SFS and control groups (*P* < 0.05). Meanwhile, VRS counts were higher in the SFS and epilepsy groups than in the control group (VRS_SFS, 442.42 ± 74.58, VRS_epilepsy, 629.94 ± 106.55, VRS_control, 354.14 ± 106.58; *P* < 0.001), and similar results were found for VRS volume (VRS_SFS, 6,228.18 ± 570.74 mm^3^, VRS_epilepsy, 9,684.84 ± 7,292.66mm^3^, VRS_control, 4,007.22 ± 118.86 mm^3^; *P* < 0.001). However, VRS metrics were lower in the SFS group than in the epilepsy group (*P* < 0.001). In both SFS and epilepsy, VRS metrics positively correlated with seizure duration and negatively correlated with the course after seizure onset.

**Conclusion:** SFS may not be associated with WM microstructural disruption; however, epilepsy is related to WM alterations. Seizures are associated with glymphatic dysfunction in either SFS or epilepsy.

## Introduction

Seizures are the most common pediatric neurologic disorders (1). Seizure episodes also cause anxiety to parents and family members. Epilepsy is defined by any of the following conditions: at least two unprovoked (or reflex) seizures occurring >24 h apart; or diagnosis of an epilepsy syndrome (2, 3). It is generally accompanied by adverse prognosis, while simple febrile seizures (SFS) are defined as generalized seizures that last for <15 min and occur once during a 24-h period in a febrile child without evidence of an intracranial infection, metabolic disturbance, or a history of afebrile seizures (4). Febrile seizures (FS), including SFS and complex febrile seizures, are widely known seizure events in childhood, affecting 2–5% of children aged 6–60 months (4, 5). SFS are estimated to account for 70–75% of FS (6). They are considered benign and self-limiting, and rarely cause long-term neurodevelopmental impairment (7). However, patients who have SFS have a slightly higher risk of developing epilepsy than other children (1 vs. 0.5%) (5, 8). Existing brain damage, a family history of seizures, or birth complications (low Apgar [appearance, pulse, grimace, activity, and respiration] scores) may contribute to the development of epilepsy following FS (9).

White matter (WM) maturation, involving the myelination of WM, in early life is critical for brain development and is influenced by perturbations from heterogeneous etiologic factors (10, 11). Previous studies suggested that FS are age-dependent responses of the immature brain to fever, and that enhanced neuronal excitability of the immature brain may result in seizures (5, 12). During the maturation process, FS may create a susceptible brain state. One study indicated that FS were associated with changes in the myelin sheath (13). Moreover, Yoong et al. demonstrated that prolonged FS caused reversible reductions in WM integrity (14). Nevertheless, in the early developmental stage, it is unclear whether the WM maturation process in children with FS is different from that in controls and whether FS can increase the susceptibility to seizure-induced brabin damage. Although SFS account for the majority of FS (6), the influence of SFS on changes in WM microstructure remains unclear. As for epilepsy, a common form of seizures, microstructural abnormalities of the WM have already been demonstrated (15, 16). However, few studies have investigated whether SFS and epilepsy show obvious variances in WM integrity. Diffusion tensor imaging (DTI) is an advanced magnetic resonance imaging (MRI) technique that can provide subtle information about tissue microstructure *in vivo* (10). Specifically, DTI-derived fractional anisotropy (FA), radial diffusivity (RD), and axial diffusivity (AD) are sensitive to the developmental progression of fiber organization and myelination in WM (17, 18). Therefore, DTI is used to explore alterations and discrepancies in WM integrity among SFS, epilepsy, and control groups.

The Virchow-Robin space (VRS) is a part of the glymphatic system and plays an important role in waste clearance from the brain, leukocyte trafficking, and immune response modulations (19). The cerebrospinal fluid (CSF) and interstitial fluid (ISF) are exchanged via the VRS and form a part of the glymphatic system circulation (19). On MRI, visible VRS was detected in children with epileptic seizures, and was related to seizure duration and the course after seizure onset (time interval between the seizure onset and MRI scan) (20, 21). This may be related to the abnormal exchange of CSF-ISF and an impaired blood–brain barrier (BBB) in epileptic seizures (22–24).

Currently, the mechanism underlying SFS remains unclear. However, previous studies have demonstrated that the inflammatory process including secretion of cytokines in the brain is associated with the generation of FS (25). Thus, the inflammatory process may impair CSF-ISF exchanges and the BBB (22). Therefore, we hypothesized that SFS may also induce changes in VRS and may be associated with seizure duration and the time interval between seizure onset and MRI scans. However, no previous study has investigated the relationship between VRS and SFS in children. Moreover, it is uncertain whether VRS alterations show discrepancies among different seizure types.

Therefore, this study aimed to investigate the changes in WM microstructures in children with SFS and epilepsy through quantitative DTI metrics. We also explored the changes of VRS using a custom-designed automated method for comparison among SFS, epilepsy, and controls.

## Materials and Methods

This retrospective study was reviewed and approved by the local institutional review board, the Clinical Research Ethics Committee of the First Affiliated Hospital of Xi'an Jiaotong University (No. XJTU1AF2015LSL-052). The parents of the children were informed of the potential risk of MRI examination, such as loud noise, and the adverse effects of oral chloral hydrate. Written informed consent was provided by the parents.

### Participants

Between May 2013 and December 2019, children aged 6–60 months who underwent MRI examinations as a part of the screening for brain disease at the Department of Radiology in the First Affiliated Hospital of Xi'an Jiaotong University were consecutively enrolled.

This study enrolled children with SFS if they met the following conditions: (i) diagnosed with SFS on the basis of the criteria defined by the American Academic of Pediatrics (4, 26); (ii) children with gestational age ≥37 weeks; (iii) no history of brain injury, head trauma, and central nervous system infections; and (iv) a time interval between seizure onset and MRI scan of <15 days. The exclusion criteria were as follows: (i) incomplete clinical information on course after seizure onset; (ii) incomplete clinical information on seizure duration; and (iii) MRI abnormalities, such as hyperintensity on T2 fluid-attenuated inversion recovery (FLAIR) (except peritrigonal terminal zone of white matter myelination that presents as a bilaterally symmetrical, slightly increased signal intensity with a hazy border on T2WI) (27).

Children who were included in the epilepsy group met the following criteria: (i) children with primary epilepsy diagnosed according to diagnostic criteria defined by the International League Against Epilepsy (3, 28); (ii) children with gestational age ≥37 weeks; (iii) no other neurological disorders, such as autism or attention deficit hyperactivity disorder; and (iv) a time interval between MRI scan and the last seizure onset before MRI scan of <15 days. The exclusion criteria were as follows: (i) incomplete clinical information on the course following seizure onset and seizure duration; (ii) a history of intracranial infection or head trauma; and (iii) MRI abnormalities, such as hyperintensity on T2 FLAIR (except peritrigonal terminal zone of white matter myelination that presents as a bilaterally symmetrical, slightly increased signal intensity with a hazy border on T2WI) (27).

Children in the control group were enrolled according to the following criteria: (i) children with gestational age ≥37 weeks; (ii) no history of epilepsy and other types of seizures; and (iii) no abnormalities on MRI. Children whose images revealed artifacts were excluded. In addition, children with neurological disorders (such as facial palsy and tic disorders) and intracranial infection were also excluded.

### MRI Data Acquisition

All participants underwent MRI examinations using the same 3.0-T scanner (Signa HDxt, GE Healthcare, Milwaukee, WI) with an 8-channel head coil at the Department of Radiology, the First Affiliated Hospital of Xi'an Jiaotong University. To reduce motion artifacts and facilitate the MRI examination, all children were adjusted while sleeping, and a relatively small dose of chloral hydrate (10%, 25–50 mg/kg) was administered on obtaining the parent's approval for infants who could not cooperate with the MRI scan (29, 30). Then, we followed up on adverse drug reactions in children who received chloral hydrate within 24 h following MRI examinations. Micro-earplugs were used to protect the child's hearing, and molded foam was used for head immobilization.

Three-dimensional fast spoiled gradient-recalled echo T1-weighted imaging (T1WI), fast spin echo (FSE) T2-weighted imaging (T2WI), T2 FLAIR, and single-shot echo-planar DTI were performed on the 3.0-T scanner. Parameters of MRI sequences (T1WI, T2WI, and T2-FLAIR) among all participants were the uniform standard. VRS was evaluated via T2WI, and T1WI and T2-FLAIR were used to differentiate VRS from other lesions. Parameters of the MRI sequences were as follows: (i) T2WI: repetition time (TR)/echo time (TE) = 4,200/120 ms; slice thickness = 4 mm without gap; FOV = 240 mm; and matrix size = 320 × 320. (ii) DTI: 30 gradient directions; b values = 0 and 600 s/mm^2^; number of b0 = 8; TR/TE =11,000/69.5 ms; slice thickness = 2.5 mm without spaces; field of vision (FOV) = 240 mm; and matrix size = 128 × 128.

### TBSS Analysis

The FMRIB software library (FSL) was used for processing DTI data. First, an eddy current correction was performed for all the subjects' DTI. Then, the brain regions were extracted using the FSL Brain Extraction Tool. The individual DTI metrics of FA, AD, and RD were calculated using the FMRIB Diffusion Toolbox (http://fsl.fmrib.ox.ac.uk/fsl/fslwiki/FDT).

Imaging processing and comparison of DTI metrics among SFS, epilepsy, and control groups were addressed using an optimized protocol as described in our previous studies (31, 32). First, group mean FA images were created from the participants in the control group. Second, images of all the participants were registered to the group mean image. A single-participant FA image with the minimum mean displacement score was selected as the final target. Finally, all individual FA images among these three groups were registered to the target using a combination of linear and nonlinear registration methods. AD and RD were normalized into the target space using the FA deformation parameters. Subsequently, WM-DTI metrics were calculated and compared among the SFS, epilepsy, and control groups. Following family-wise error (FWE) rate correction and threshold-free cluster enhancement (TFCE), *P* < 0.05 was considered to indicate statistical significance.

### VRS Segmentation

A custom script was written using MATLAB (R2012b; MathWorks, Natick, MA) to automate the segmentation of VRS in the WM above the bilateral ventricles (including the bilateral ventricular level) (33, 34). In this segmentation process, the skulls were removed using a custom software built with ITK (https://www.itk.org). The images were subsequently processed to obtain the WM, gray matter, and cerebrospinal fluid regions using the FMRIB software library (FSL) (https://fsl.fmrib.ox.ac.uk/fsl/fslwiki). Then, a custom-designed method in MATLAB was used to quantitate the VRS via the images exported from FSL. The core of the algorithm used to segment visible VRS included two-dimensional (2D) Frangi filtering which enables the filtering of vessel-like tubular structures from 2D images of axial plane T2 images (34). Subsequently, the brain volume, WM volume, VRS count, and VRS volume were obtained based on the segmentation.

### Statistical Analysis

The Mann Whitney U test was used to compare demographic data among the SFS, epilepsy, and control groups. Differences in VRS counts and volume among the three groups were also analyzed by the Mann Whitney U test. Pearson correlations were applied to investigate the relationships of VRS counts and volume with seizure duration and course after seizure onset. SPSS software (Version 21.0; IBM, Armonk, New York, USA) was used for statistical analysis. According to the Bonferroni correction, *P* < 0.017 was indicated as statistically significant while multiple comparisons were carried out among the three groups.

## Results

### Study Population

**Figure 1:**
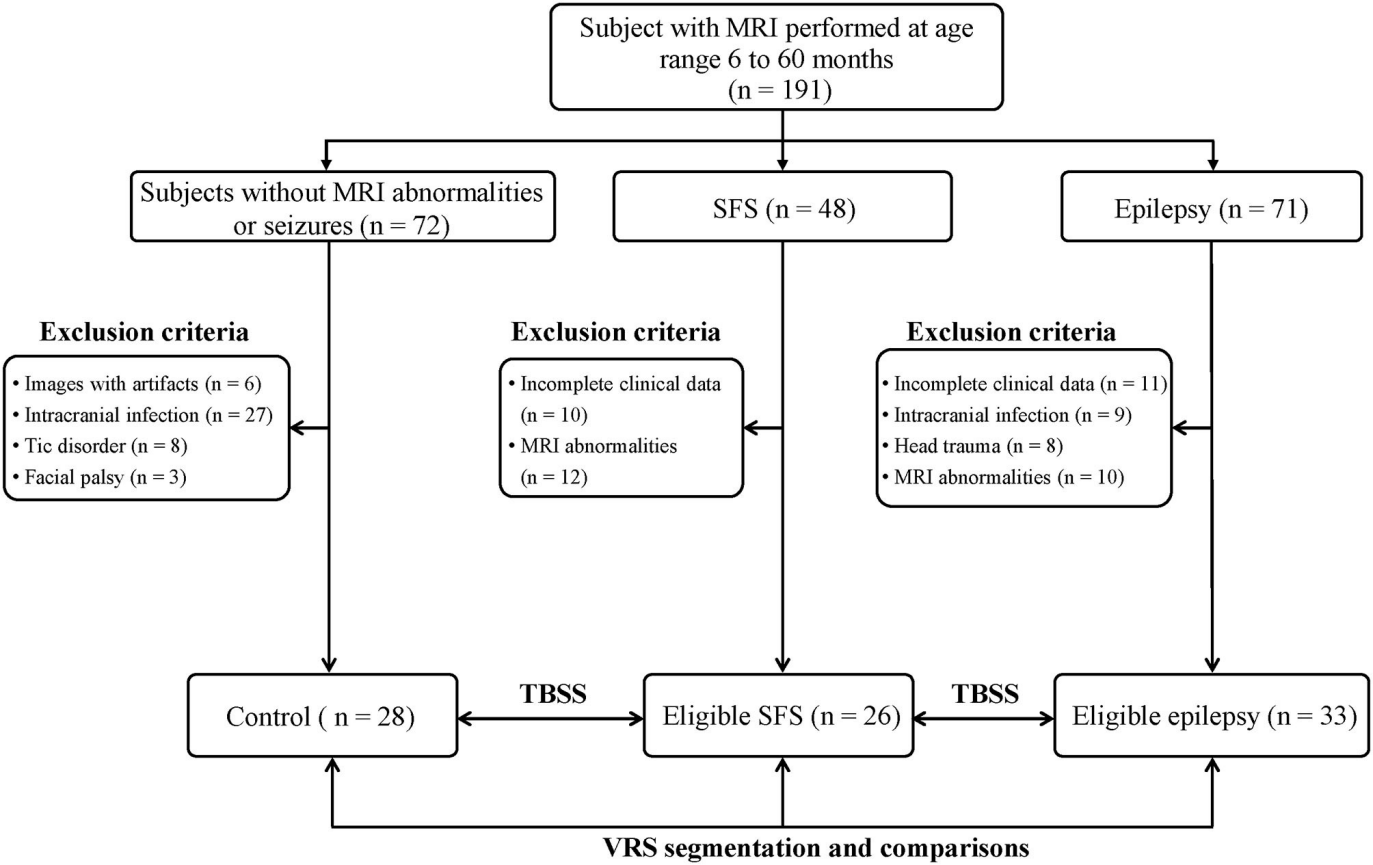
Participant flow chart based on inclusion and exclusion criteria.

Based on the inclusion and exclusion criteria, the study finally enrolled 87 participants: 26 in the SFS group, 33 in the epilepsy group, and 28 in the control group (Figure 1). There were no significant differences in age, sex, and gestational age among the SFS, epilepsy, and control groups (Table 1). For the epilepsy group, all subjects were newly diagnosed without a history of anti-epilepsy medications and 10 had focal epilepsy and 23 had generalized epilepsy.

**Table 1:** Demographics for the SFS, epilepsy, and control groups.

	**SFS (*n* = 26)**	**Epilepsy (*n* = 33)**	**Control (*n* = 28)**	***P*****-value**		
				**SFS vs. control**	**Epilepsy vs. control**	**SFS vs. epilepsy**
Age (months)^a^	26.92 ± 12.39	28.88 ± 12.14	25.75 ± 12.23	0.938	0.994	0.897
Gender (male)	16 (61.54%)	21 (63.63%)	17 (60.71%)	0.951	0.816	0.870
GA (weeks)^a^	39.35 ± 1.23	39.21 ± 1.08	39.32 ± 1.36	1	0.515	0.487
Seizure duration (minutes)^a^	2.54 ± 1.45	5.61 ± 2.59	NA	NA	NA	<0.001
Course after seizure onset (days)^a^	3.69 ± 1.38	3.42 ± 1.43	NA	NA	NA	0.427

a *mean ± standard deviation*.

*Course after seizure onset, the time interval between seizure onset and MRI scan*.

*NA, Not applicable*.

### White Matter Analysis Based on TBSS

**Figure 2:**
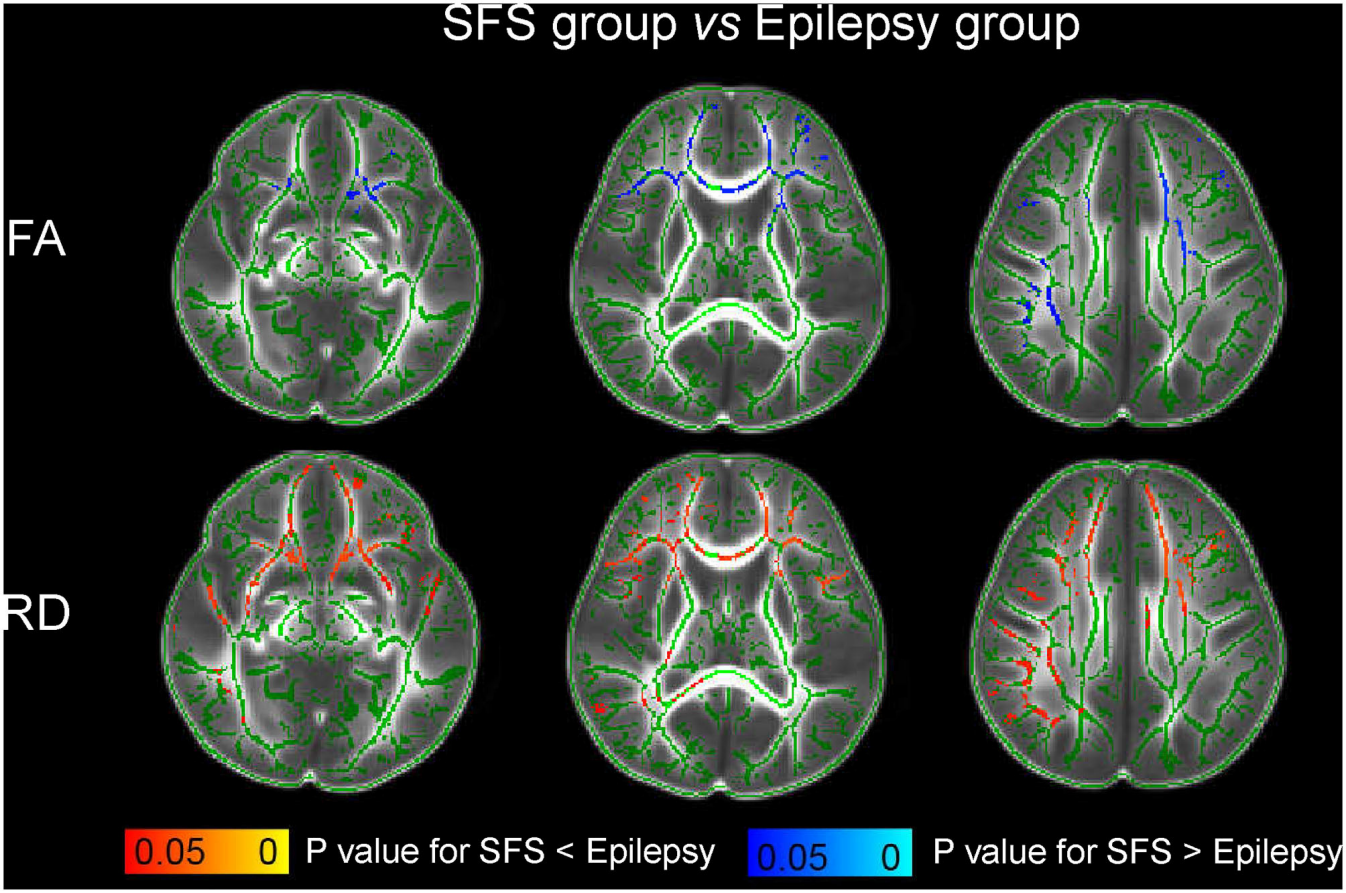
Comparison of white matter-DTI metrics between the SFS and epilepsy groups based on TBSS. The green color indicates the fibrous skeleton of brain white matter and represents no significant difference. The red-yellow color scale and blue-light blue color scale indicates significant changes of DTI metrics (*P* < 0.05).

**Figure 3:**
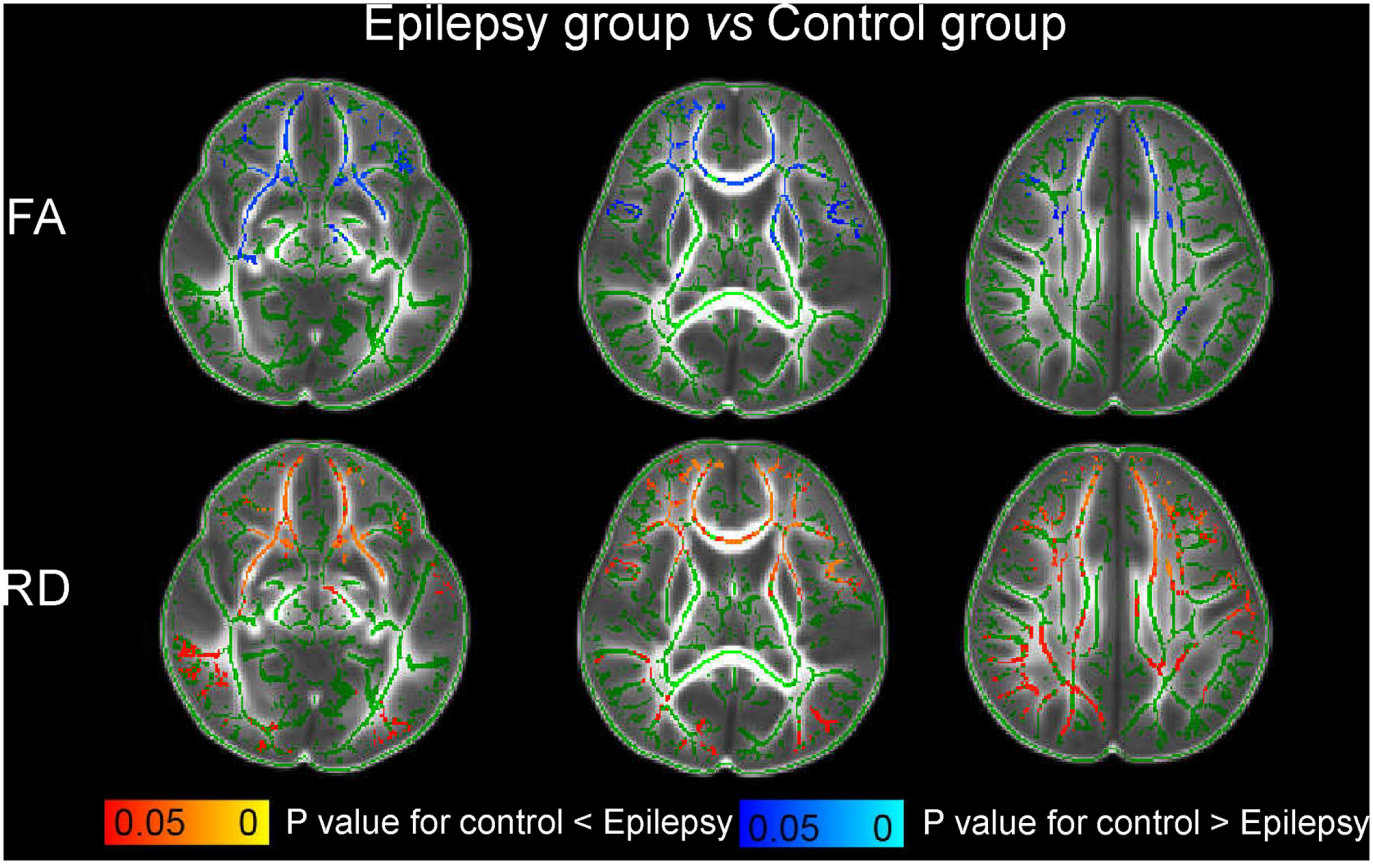
Comparison of white matter-DTI metrics between the epilepsy and control groups based on TBSS. The green color indicates the fibrous skeleton of brain white matter and represents no significant difference. The red-yellow color scale and blue-light blue color scale indicates the significant changes of DTI metrics (*P* < 0.05).

In comparison with the control group, the children with SFS showed no significant changes of FA, AD, or RD in WM (*P* > 0.05). However, compared to the children with SFS, those with epilepsy showed decreased FA, unchanged AD, and increased RD (Figure 2). Significant changes were mainly observed in the genu of corpus callosum (GCC), bilateral anterior thalamic radiation (ATR), inferior frontal-occipital fasciculus (IFOF), inferior longitudinal fasciculus, superior longitudinal fasciculus, and uncinate fasciculus. More WM regions showed significantly increased RD than decreased FA. Meanwhile, comparing WM-DTI metrics between epilepsy groups and controls, decreased FA, unchanged AD, and increased RD values were also found in children with epilepsy (Figure 3), and similarly significant regions were observed in comparisons between the SFS and epilepsy groups.

### Comparisons of VRS Findings Among Children With SFS, Epilepsy, and Controls

**Figure 4:**
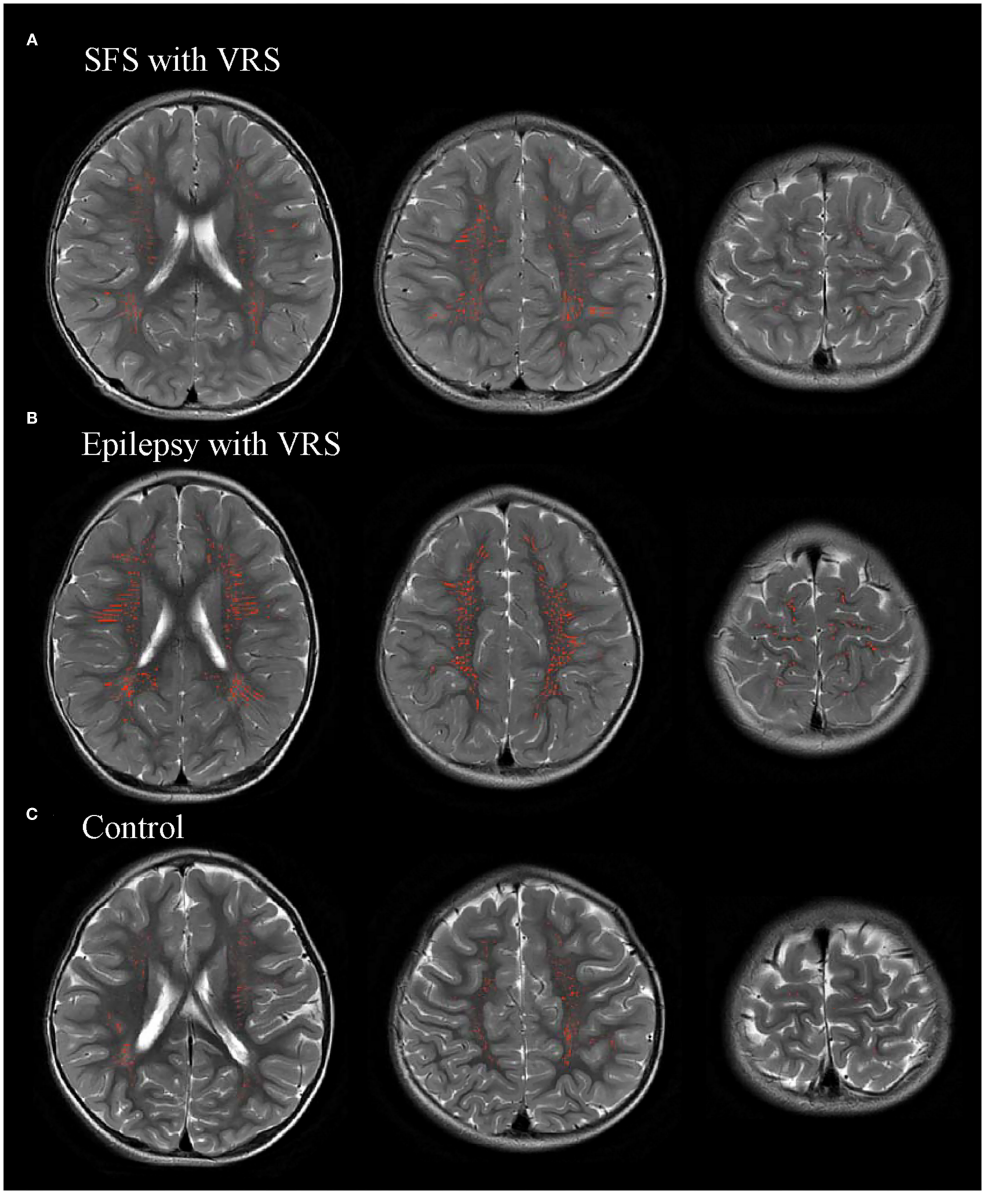
Representative images of automatic segmentation of the visible Virchow-Robin space (VRS).

VRS counts and volume were quantified using an automated segmentation method in children with SFS, children with epilepsy, and the controls (Figure 4). There were significant differences in VRS counts and volume among the SFS, epilepsy, and control groups (*P* < 0.001). The counts and volume of visible VRS were significantly higher in the SFS and epilepsy groups than that in the control group (*P* < 0.001). However, visible VRS counts and volume in the SFS group were lower than those in the epilepsy group (*P* < 0.001). The brain and WM volumes were not significantly different among the three groups (*P* > 0.017) (Table 2).

**Table 2:** Comparisons of VRS among the SFS, epilepsy, and control groups.

	**SFS (*n* = 26)**	**Epilepsy (*n* = 33)**	**Control (*n* = 28)**	***P*****-value**		
				**SFS vs. control**	**Epilepsy vs. control**	**SFS vs. epilepsy**
VRS counts	442.42 ± 74.58	629.94 ± 106.55	354.14 ± 106.58	<0.001	<0.001	<0.001
VRS volume (mm^3^)	6,228.18 ± 570.74	9,684.84 ± 7,292.66	4,007.22 ± 118.86	<0.001	<0.001	<0.001
WMV (×10^3^ mm^3^)	312.85 ± 68.80	295.42 ± 57.40	312.31 ± 34.67	0.416	0.325	0.843
BV (×10^3^ mm^3^)	894.94 ± 86.51	878.48 ± 82.00	912.87 ± 88.20	0.591	0.132	0.252

*WMV, white matter volume; BV, brain volume*.

**Figure 5:**
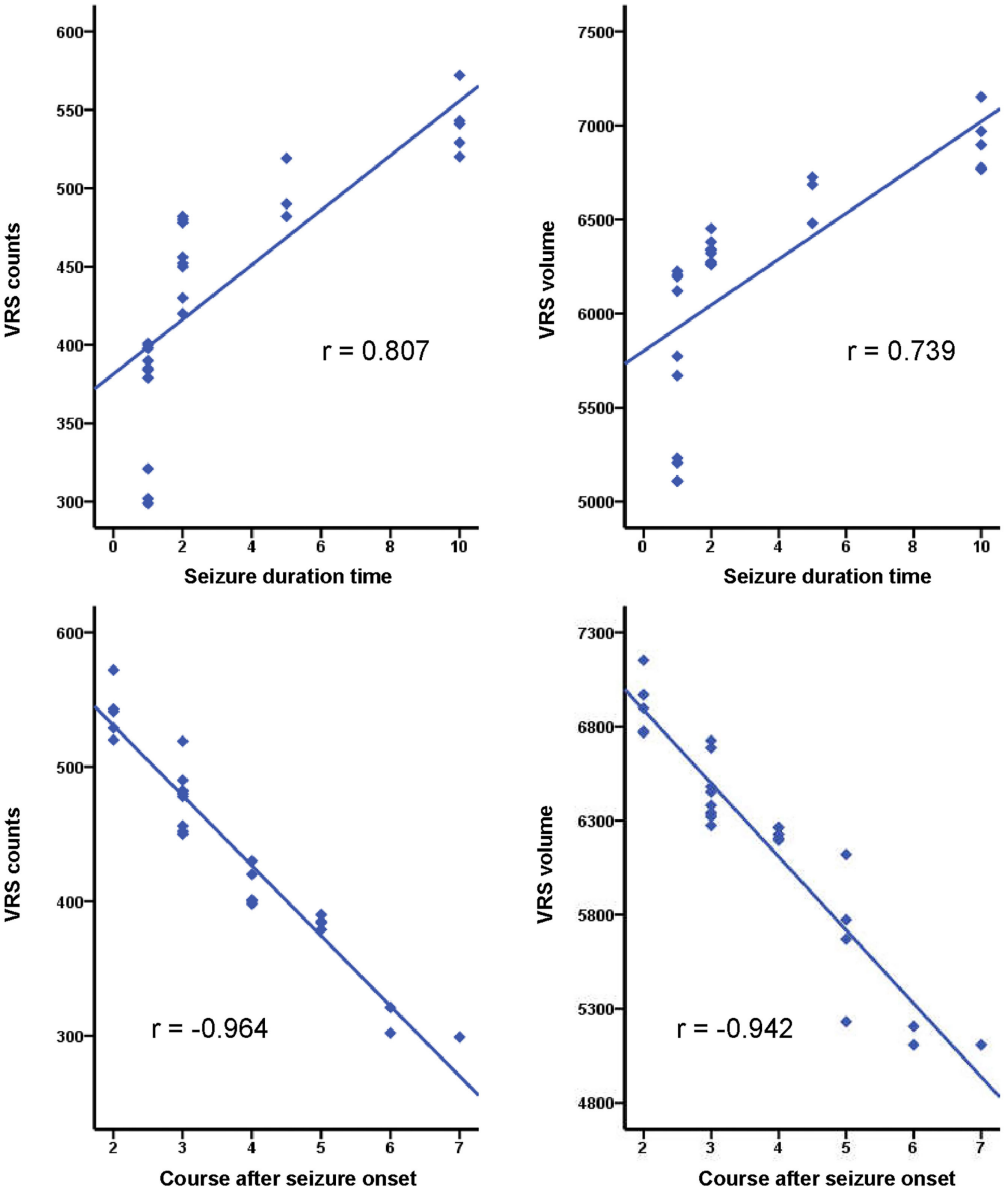
Correlations of VRS metrics and seizure duration time, and course after seizure onset in the SFS group (*P* < 0.001).

**Figure 6:**
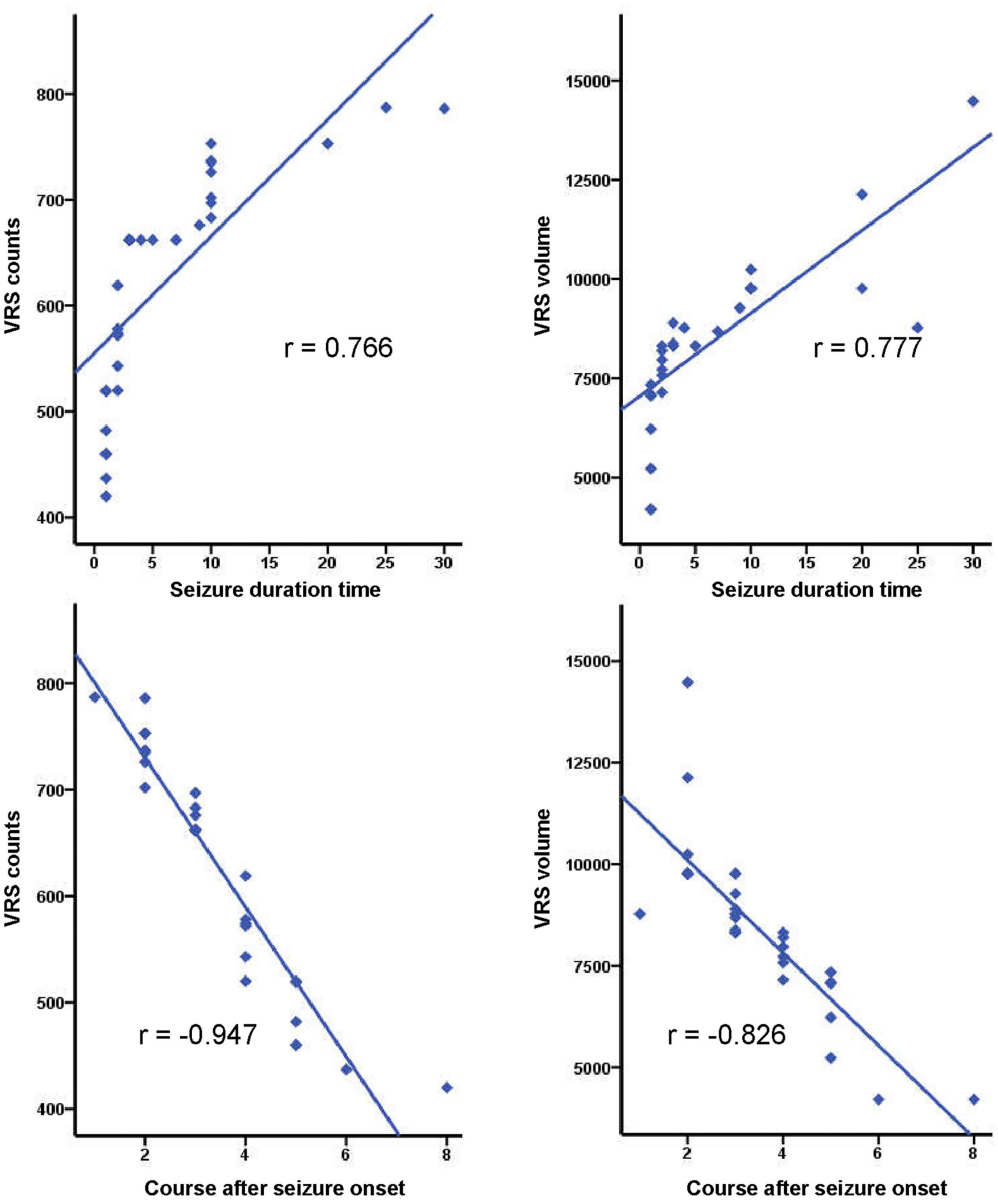
Correlations of VRS metrics and seizure duration time, and course after seizure onset in the epilepsy group (*P* < 0.001).

As for correlations between VRS metrics and seizure duration, and the course after seizure onset in the SFS and epilepsy groups, positive correlations were observed between seizure duration and the VRS counts, and VRS volume in both SFS and epilepsy groups (SFS: r__vol_ = 0.739, r__count_ = 0.807, *P* < 0.001; epilepsy: r__vol_ = 0.777, r__count_ = 0.766, *P* < 0.001). In contrast, the course after seizure onset showed negative correlations with both VRS counts and VRS volume in both groups (SFS: r__vol_ = −0.942, r__count_ = −0.964, *P* < 0.001; epilepsy: r__vol_ = −0.826, r__count_ = −0.947, *P* < 0.001) (Figures 5, 6).

## Discussion

In this study, we evaluated WM microstructure changes in children with SFS and epilepsy in comparison with controls using TBSS. No significant changes of FA, AD, or RD were found in children with SFS in comparison with controls. However, in comparison with the control and SFS groups, the epilepsy group showed decreased FA, unchanged AD, and increased RD. Meanwhile, we also quantified the visible VRS counts and volume in the three groups and explored the relationships among seizure duration, course after seizure onset, and VRS metrics. VRS metrics were higher in the SFS and epilepsy groups than in the controls. But VRS metrics were lower in children with SFS than in those with epilepsy. Positive correlations of VRS metrics with seizure duration and negative correlation of VRS metrics with the course after seizure onset were found in both SFS and epilepsy groups.

TBSS has recently been identified as a general approach for voxel-wise analysis of diffusion data along the WM skeleton, which can provide subtle information on WM microstructure (35). Based on the voxel-wise analysis of DTI, our results showed that DTI metrics did not change in the WM of the SFS group compared to the controls, while children with epilepsy presented with decreased FA, unchanged AD, and increased RD compared with SFS or the controls. Few systematic studies have investigated WM integrity associated with diffusion metrics in children with SFS. Combinations of unchanged AD and decreased RD with age could reflect the myelination of WM (17, 36, 37). In comparison with the controls, our study found no changes of AD or RD in SFS. This suggested that SFS may not induce WM alterations. But these findings are not consistent with the results of another study in which myelin water fraction (MWF) changes were observed in FS (higher MWF in SFS, lower MWF in complex FS) (13). Although increased MWF indicated more myelin in these patients, it may not be representative due to the small sample size (3 SFS, 4 complex FS). At present, more studies have focused on complex FS and prolonged FS. Decreased FA and increased RD were found in children with prolonged FS at 1 and 6 months post prolonged FS (14, 38). However, it was observed that damaged WM integrity caused by prolonged FS was reversible (14). SFS are mild febrile seizures compared with prolonged FS. Therefore, SFS may plausibly not result in a reduction of WM integrity. Additionally, one study assessed the relationship with temporal lobe epilepsy in adults who had experienced SFS and demonstrated that SFS were not associated with temporal lobe epilepsy and did not cause prolonged WM changes (39). These findings were consistent with our results.

In our study, decreased FA and increased RD were observed in children with epilepsy compared with SFS and controls. The results indicated that WM integrity of children with epilepsy was damaged or myelination was delayed, which was consistent with the results of previous studies (40–42). Other studies also showed significantly changed regions such as GCC, ATR, and IFOF (15, 40). Combined with previous research, the findings suggested that impaired WM integrity with abnormal myelin and fiber organization was present in children with new-onset epileptic seizures and may precede the onset of epilepsy (43). Contrary to the findings for children with epilepsy, our results further validated that SFS could not induce changes in WM.

The cerebral glymphatic system, particularly the VRS, plays an important role in CSF-ISF circulation and waste clearance from the brain (19, 44). A previous study suggested that epileptic seizures were associated with dilated VRS (45). We quantified the VRS in controls and in children with SFS and epilepsy via a custom-designed automated method, using the same MRI processing method as that employed in a previous study (45). Our results showed that the VRS counts and volume were higher in the SFS and epilepsy groups than in controls that were in line with previous findings (45). The increased visible VRS in children with SFS and epilepsy may be associated with seizures and cerebral glymphatic circulation. Although the mechanism underlying FS remains unclear, the inflammatory process is an important part of both FS and epileptic seizures (23, 46–48). Proinflammatory mediators can induce BBB damage by affecting the endothelial tight junctions and the basal membrane (23, 49). Infiltrating leukocytes and albumin would accumulate in the VRS through the damaged BBB, resulting in dilation of the VRS on MRI. In addition, BBB disruption may contribute to abnormal CSF-ISF circulation (22). CSF-ISF exchange occurs via the VRS and forms part of the glymphatic system circulation (19). Disruption of CSF-ISF exchange can hinder clearance of waste and secretion products, aggravating VRS dilation (50). Consistent with these findings, our study observed increased VRS counts and volume in children with seizures, either FS or epilepsy.

Nevertheless, the VRS counts and volume were lower in the children with SFS than in those with epilepsy. SFS are a type of mild seizure with a short duration (<15 min) and do not usually recur within the next 24 h (5), unlike epilepsy. A previous study suggested that seizure severity was related to dysfunction of the glymphatic system (45). Our study also consistently showed that VRS metrics positively correlated with seizure duration and negatively correlated with the course after seizure onset. Thus, it is plausible that SFS caused lower VRS counts and volume with respect to epilepsy. Therefore, our study revealed that dysfunction of the glymphatic system may be caused by seizures and that the degree of this dysfunction may be related to seizure severity. In addition, a previous study showed that an increasing perivascular space was associated with and may result in WM damage (51). Considering that SFS has less severe seizures than epilepsy, we speculate that changes in VRS metrics in SFS may not induce WM alterations. Glymphatic dysfunction may be a precursor of seizure-induced brain damage. However, better research designs are warranted for demonstrating the association between WM integrity and the glymphatic system.

There were several limitations in our study. First, this was a cross sectional study with a limited sample size. We did not follow-up children with SFS or investigate the risk factors for progression of SFS to epilepsy. However, our findings showed that SFS could not cause WM microstructural damage post-onset, which may help address the concerns of parents with children presenting with SFS. Second, clinical data, such as cytokine levels, were not measured in patients with SFS and epilepsy. These data may further validate the relationships between seizures and glymphatic function.

## Conclusion

In conclusion, SFS cannot cause WM microstructural disruptions or delay WM development; but epileptic seizures are related to WM alteration. Both SFS and epilepsy are associated with dysfunction of the glymphatic system, and the changes in VRS are related to the seizure duration and the course after seizure onset.

## Data Availability Statement

The datasets presented in this article are not readily available because application is required to be available by request. Requests to access the datasets should be directed to Jian Yang, yj1118@xjtu.edu.cn.

## Ethics Statement

The studies involving human participants were reviewed and approved by the First Affiliated Hospital of Xi'an Jiaotong University. Written informed consent to participate in this study was provided by the participants' legal guardian/next of kin.

## Author Contributions

MSa and CL collaboratively acquired the MRI data of all the participants, and then processed all the data, and wrote and revised the preliminary manuscript together. XL and JY conducted this work. SS and ML helped process the DTI data together. MSi, HT, and AP participated in the VRS processing. YC, XW, FW, YZ, and HZ collaboratively acquired the clinical data of all the participants. GY designed the custom automated-method of VRS processing. All authors contributed to the article and approved the submitted version.

## Conflict of Interest

The authors declare that the research was conducted in the absence of any commercial or financial relationships that could be construed as a potential conflict of interest.
